# Construct validity and reliability of the perceived stress scale for nursing students in South Africa

**DOI:** 10.4102/curationis.v45i1.2276

**Published:** 2022-06-06

**Authors:** Michelle C. Engelbrecht

**Affiliations:** 1 Centre for Health Systems Research & Development, Faculty of Humanities, University of the Free State, Bloemfontein, South Africa

**Keywords:** perceived stress scale, nursing students, construct validity, reliability, South Africa

## Abstract

**Background:**

Increased levels of stress in nursing students are negatively related to caring behaviours and also result in poorer job proficiency and nurses who are more inclined to leave the profession. The Perceived Stress Scale (PSS), developed by Sheu and colleagues, is one of the most cited instruments for measuring stress and sources of stress amongst nursing students in international studies. However, it has not been widely validated for this purpose.

**Objectives:**

This research aimed to test the construct validity and reliability of the PSS for South African nursing students.

**Method:**

A cross-sectional survey was conducted at a Central South African University, and 471 of the 685 registered nursing students (68.8% response rate) participated in the study. Questionnaires were distributed and collected during classes. Confirmatory factor analysis was performed to test the hypothesised six-factor latent structure and determine the construct validity of the PSS. The internal consistency of the PSS was measured using Cronbach’s alpha.

**Results:**

The model fit was a good fit and supported the six-factor latent structure as stress from (1) taking care of patients, (2) teachers and nursing staff, (3) assignments and workload, (4) peers and daily life, (5) lack of professional knowledge and skills and (6) clinical environment. Overall the PSS had a Cronbach’s alpha of 0.93.

**Conclusion:**

The results confirm the construct validity and the internal consistency of the PSS for South African nursing students.

## Introduction

Students in higher education face increasing levels of stress, particularly academic-related stress (Mason 2017:138; Pascoe, Hetrick & Parker 2020:104–112; Ribeiro et al. 2018:70–77). In comparison to the general student population, nursing students experience even higher levels of stress (Bartlett, Taylor & Nelson 2016:89), which may be largely due to the nature of nursing education (Gomathi, Jasmindebora & Baba 2017:108). The nursing curriculum comprises a theoretical and practical component, which commences during the first year of study. Busy timetables, examinations requiring critical thinking, as well as clinical practice are just some of the demands faced by nursing students (Aedh, Elfaki & Mohamed 2015:34–36). The sources of stress of nursing students can be broadly categorised as clinical, academic, and personal. They include stress from lack of professional knowledge, clinical environment, taking care of patients, assignments and workload, teachers and nursing staff, peers and daily life (Aedh et al. 2015:34–36; Ahmed & Mohammed 2019:119; Alsaqri 2017:3; Hamaideh, Al-Omari & Al-Modallal 2017:200; Karaca et al. 2017:34; Khater, Akhu-Zaheya & Shaban 2014:198; Labrague 2013:427–428; Labrague et al. 2018:404–405; Mohamed & El-Hafez 2015:42; Ugwoke et al. 2018:13214–13215), long working hours, poor study methods (Langtree, Razak & Haffejee 2018:92), poor grades, inability to balance work and leisure, difficulty in balancing clinical work and studying and the humiliating behaviour of doctors (Parveen & Inayat 2017:3–4).

According to Lazarus and Folkman (1984):

[*P*]sychological stress is a particular relationship between the person and the environment that is appraised by the person as taxing or exceeding his or her resources and endangering his or her well-being. (p. 19)

Stress experienced by higher education students, including nursing students, impacts their mental and physical health and may lead to a range of academic (Pascoe et al. 2020:108) and social problems (Gomathi et al. 2017:108; Ribeiro et al. 2018:75–76).

Whilst stress amongst nurses has been well studied, there are few instruments to measure stress amongst nursing students specifically (Watson et al. 2013:160). Systematic reviews of studies conducted from 2000 to 2015 (Labrague et al. 2017:471–480) and 2010–2016 (McCarthy et al. 2018:197–209) on stress in nursing students revealed that the majority of the studies utilised the Perceived Stress Scale (PSS) developed by Sheu et al. (1997:341–351). The PSS was developed to determine the type of stress perceived and levels of stress experienced in clinical settings by nursing students at Taiwanese Universities. Whilst the PSS has been widely used internationally (i.e. Europe and Asia), previous studies have not fully examined the psychometric properties of the scale (Algaralleh, Altwalbeh & Alzayyat 2019:778). Furthermore, there are limited African studies that have utilised this instrument, and neither tested the factor structure of the scale (Labrague et al. 2018:402–408; Ugwoke et al. 2018:13212–13218). In particular, Langtree et al. (2018:91) reported developing a new questionnaire to identify the causes of stress in first-year South African nursing students, as there was not an appropriate research instrument for their study. With this in mind, the paper aims to test, for the first time, the hypothesised six-factor latent structure and establish the construct validity and internal consistency of the PSS for South African nursing students.

## Research methods and design

### Design, setting and sample

A cross-sectional survey, which collects data at one point in time, was undertaken at a purposively selected South African University. Purposive sampling relies on the researcher’s knowledge and judgment of the context. In this regard, the first author was familiar with the purposively selected university and was able to obtain permission for the study and access to respondents with relative ease (Babbie 2016:106, 187). At the time of the study, there were 685 registered nursing students at the university. A total of 471 questionnaires were completed with a 68.8% response rate, of which 27 had extensive missing data and were discarded, leaving a total of 444 questionnaires.

### Data collection

Data collection took place early in October 2018. In collaboration with the class facilitator, fieldworkers distributed envelopes containing information leaflets, consent forms and questionnaires to the students at the start of their class. Interested students were allowed time during class to complete the questionnaire. In order to maintain confidentiality, signed consent forms and completed questionnaires were placed in a sealed envelope and returned to the class facilitator. The research team separated the consent forms from the questionnaires so that no information could be linked to a particular respondent.

### Measures

The questionnaire comprised demographic and background questions as well as the PSS. The demographic and background questions focussed on issues such as gender, age, marital status, dependents, home language, place of residence, source of payment of university fees and year of study (Engelbrecht & Wilke 2021:141). The PSS (Sheu et al. 1997:341–351) includes 29 items measured on a five-point Likert Scale ranging from 0 (Never) to 4 (Very often). Factor analyses in previous samples have shown a six-factor model for the PSS, consisting of stress from (1) taking care of patients, (2) teachers and nursing staff, (3) assignments and workload, (4) peers and daily life, (5) lack of professional knowledge and skills and (6) clinical environment. Table 1 provides an indication of which items were hypothesised to measure the six factors identified in earlier research (Sheu et al. 1997:341–351; Sheu, Lin & Hwang 2002:165–175).

**TABLE 1:** Factor structure of the perceived stress scale†.

Factors	Items
**Stress fr om taking care of patients**	PSS_Q1 Lack of experience and ability in providing nursing care and making judgments
	PSS_Q2 Not knowing how to help patients with physio-psycho-social problems
	PSS_Q3 Not being able to reach one’s expectations
	PSS_Q4 Not being able to provide appropriate responses to doctors’, teachers’, and patients’ questions
	PSS_Q5 Worry about not being trusted or accepted by patients or patients’ families
	PSS_Q6 Not being able to provide patients with good nursing care
	PSS_Q7 Not knowing how to communicate with patients
	PSS_Q8 Difficulties in changing from the role of student to that of a nurse
**Stress from assignments and workload**	PSS_Q9 Worry about bad grades
	PSS_Q10 Pressure from the nature and quality of clinical practice
	PSS_Q11 Feeling one’s performance does not meet teachers’ expectations
	PSS_Q12 Feeling the requirements of clinical practice exceeds one’s physical and emotional endurance
	PSS_Q13 Dull and inflexible clinical practice affects one’s family and social life
**Stress from lack of professional knowledge**	PSS_Q14 Unfamiliar with medical history and terms
	PSS_Q15 Unfamiliar with professional nursing skills
	PSS_Q16 Unfamiliar with patients’ diagnoses and treatments
**Stress from the environment**	PSS_Q17 Stress in the hospital environment where clinical practice takes place
	PSS_Q18 Unfamiliar with ward facilities
	PSS_Q19 Stress from rapid change in patient’s condition
**Stress from peers and daily life**	PSS_Q20 Competition from peers in school and clinical practice
	PSS_Q21 Pressure from teachers who evaluate students’ performance by comparison
	PSS_Q22 Clinical practice affects involvement in extracurricular activities
	PSS_Q23 Cannot get along with peers in the group
**Stress from teachers and nursing staff**	PSS_Q24 Discrepancy between theory and practice
	PSS_Q25 Not knowing how to discuss patient’s illness with teachers or medical and nursing personnel)
	PSS_Q26 Teacher’s instruction is different from one’s expectations
	PSS_Q27 Doctors lack empathy and are not willing to help
	PSS_Q28 Teachers do not give fair evaluation of students
	PSS_Q29 Lack of care and guidance from teachers

*Source*: Sheu, S., Lin, H., Hwang, S., Yu, P., Hu, W. & Lou, M., 1997, ‘The development and testing of perceived stress scale of clinical practice’, *Nursing Research* 5(4), 341–351 and Sheu, S., Lin, H.S. & Hwang, S.L., 2002, ‘Perceived stress and physio-psycho-social status of nursing students during their initial period of clinical practice: The effect of coping behaviors’, *International Journal of Nursing Studies* 39, 165–175. https://doi.org/10.1016/s0020-7489(01)00016-5

† , PSS, perceived stress scale.

Higher scores indicate higher levels of stress. Usually, both total scores and individual subscale scores are measured. The cut-off points for levels of perceived stress are as follows: high 2.67–4.00, moderate 1.34–2.66 and low 0.0–1.33 (Alsaqri 2017:3; Labrague 2013:426).

Reliability of the PSS has been found to be high in various studies, with Cronbach’s alpha ranging from 0.87 to 0.93 (Alsaqri 2017:3; Hamaideh et al. 2017:199; Khater et al. 2014:196; Sheu et al. 2002:168; Ugwoke et al. 2018:13214). A content validity index of 0.94 was also reported. In addition, 50.7% of the total variance was accounted for by the six factors, which confirmed the construct validity of the scale (Sheu et al. 2002:168).

### Data analysis

Statistical Package for the Social Sciences (SPSS) (IBM SPSS version 25) and R program version 3.6.0 (R Core Team 2016) and version 0.5–23, of the lavaan package (Rosseel 2012:1–36) were used for data analysis. Categorical variables were described using frequencies and percentages, and means (M) and standard deviations (s.d.) were calculated for continuous variables. Composite scores were calculated for the subscales. The internal consistency of the scale and subscales was tested using Cronbach’s alpha.

Confirmatory factor analysis was used to test whether the data supports a hypothesised model and is suitable when the theoretical constructs are well understood. This study tested the six-factor model proposed by Sheu et al. (1997:341–351, 2002:165–175). Confirmatory factor analysis requirements of multicollinearity, residual values and multivariate outliers and normality were examined. A weighted-least squares estimator with robust estimation of means and variances (WLSMV) was used; the comparative fit index (CFI) and the Tucker-Lewis Index (TLI) confirmed if the model fitted the data; the root mean square error of approximation (RMSEA) measured whether the model represented the data patterns. The performance of the model was tested by examining differences between the expected and actual correlation matrix (Kigozi 2020:3).

### Ethical considerations

The Ethics Committee at the Faculty of Economic and Management Sciences, University of the Free State (UFS-HSD2017/1097), provided ethical clearance for the study. Voluntary, informed consent was obtained from all respondents.

## Results

### Biographic characteristics

Four out of five respondents, 88.5%, were female and their average age was 28.4 years (s.d.: 9.94). Slightly less than half of the respondents were married or in a long-term relationship (46.5%), and 38.7% had children who were financially dependent on them (see Table 2 for biographic information).

**TABLE 2:** Biographic information.

Characteristics	*N*	%
**Gender** (*N* = 444)		
Male	51	11.5
Female	393	88.5
**Married or in a long-term relationship** (*N* = 440)	203	46.5
**Financially dependent children** (*N* = 444)	172	38.7
**Place of residence** (*N* = 443)		
At home with family	180	40.6
Student house off-campus	127	28.7
Rent accommodation off-campus	75	16.9
Residence on campus	61	13.8
**Payment of university fees**(*N* = 438)		
Bursary	154	35.2
Pay myself	135	30.8
Parents pay	94	21.5
Student loan	55	12.6
**Year of study**		
Undergraduate	252	56.8
Postgraduate	192	43.2

### Internal consistency

A Cronbach’s alpha value of 0.7 suggests that a scale has adequate internal consistency (Taber 2018:1278). Overall, the PSS had a Cronbach’s alpha of 0.93. As can be seen in Table 3, all subscales had a Cronbach’s alpha of at least 0.7.

**TABLE 3:** Internal consistency of the perceived stress scale†.

Perceived stress scale	Items	Cronbach’s alpha
Stress from taking care of patients	8	0.80
Stress from assignments and workload	5	0.85
Stress from lack of professional knowledge and skills	3	0.92
Stress from the environment	3	0.70
Stress from peers and daily life	4	0.71
Stress from teachers and nursing staff	6	0.81

**Total scale**	**29**	**0.93**

† , PSS, perceived stress scale.

### Construct validity

Descriptive statistics for all observed variables are provided in Table 4.

**TABLE 4:** Descriptive statistics for observed variables.

Scales and variables†	Mean	s.d.	Min‡	Max§
**Stress from a lack of professional knowledge:**	1.06	0.86	0	4
PSS_Q14 Unfamiliar with medical history and terms	1.13	0.91	0	4
PSS_Q15 Unfamiliar with professional nursing skills	0.92	0.91	0	4
PSS_Q16 Unfamiliar with patients’ diagnoses and treatments	1.14	0.95	0	4
**Stress from assignments and workload:**	2.23	0.96	0	4
PSS_Q9 Worry about bad grades	2.63	1.33	0	4
PSS_Q10 Experience pressure from the nature and quality of clinical practice	2.17	1.16	0	4
PSS_Q11 Feel that one’s performance does not meet teachers’ expectations	2.14	1.20	0	4
PSS_Q12 Feel that the requirements of clinical practice exceed one’s physical and emotional endurance	2.09	1.16	0	4
PSS_Q13 Feel that dull and inflexible clinical practice affects one’s family and social life	2.14	1.19	0	4
**Stress from taking care of patients:**	1.14	0.62	0	4
PSS_Q1 Lack of experience and ability in providing nursing care and in making judgments	1.3	0.91	0	4
PSS_Q2 Do not know how to help patients with physio-psycho-social problems	1.44	1.02	0	4
PSS_Q3 Unable to reach one’s expectations	1.43	0.89	0	4
PSS_Q4 Unable to provide appropriate responses to doctors’, teachers’, and patients’ questions	1.26	0.92	0	4
PSS_Q5 Worry about not being trusted or accepted by patients or patients’ families	1.13	1.02	0	4
PSS_Q6 Unable to provide patients with good nursing care	0.68	0.81	0	4
PSS_Q7 Do not know how to communicate with patients	0.74	0.99	0	4
PSS_Q8 Experience difficulties in changing from the role of a student to that of a nurse	1.14	1.10	0	4
**Stress from the clinical environment:**	1.36	0.76	0	4
PSS_Q17 Feel stressed in the hospital environment where clinical practice takes place	1.28	0.94	0	4
PSS_Q18 Unfamiliar with the ward facilities	1.05	0.92	0	4
PSS_Q19 Feel stressed from the rapid change in patient’s condition	1.76	1.08	0	4
**Stress from teachers and nursing staff:**	1.39	0.78	0	4
PSS_Q24 Experience discrepancy between theory and practice	1.45	1.02	0	4
PSS_Q25 Do not know how to discuss patients’ illness with teachers or medical and nursing personnel	0.90	0.94	0	4
PSS_Q26 Feel stressed that teacher’s instruction is different from one’s expectations	1.57	1.16	0	4
PSS_Q27 Doctors lack empathy and are not willing to help	1.75	1.15	0	4
PSS_Q28 Feel that teachers do not give fair evaluation of students	1.44	1.16	0	4
PSS_Q29 Lack of care and guidance from teachers	1.26	1.15	0	4
**Stress from peers and daily life:**	1.71	0.86	0	4
PSS_Q20 Experience competition from peers in school and clinical practice	1.67	1.18	0	4
PSS_Q21 Feel pressure from teachers who evaluate students’ performance by comparison	1.91	1.29	0	4
PSS_Q22 Feel that clinical practice affects one’s involvement in extracurricular activities	2.27	1.25	0	4
PSS_Q23 Cannot get along with other peers in the group	1.01	0.98	0	4
**Perceived stress scale**	1.48	0.61	0	4

† , PSS, perceived stress scale;

‡ , 0 = Never;

§ , 4 = Very often.

Exploratory data analysis revealed that the assumptions of multi-collinearity, residual values and multivariate outliers were met. However, there were substantial deviations from normality for some of the variables, which was not surprising given the ordinal nature of the data. Diagonally weighted least squares estimation was used to account for the violation of the normality assumption. The latent factors were standardised, allowing free estimation of all factor loadings. The model fit was good, with a TLI of 0.937, a CFI of 0.943, and an RMSEA of 0.075 90% confidence interval (CI) (0.071, 0.08). As expected, the indicators all showed significant positive factor loadings, with standardised coefficients ranging from 0.513 to 0.966 (see Table 5).

**TABLE 5:** Factor loadings.

Latent factor (stress from)*	Indicator	*B*	s.e.	*Z*	Beta	Sig.
Lack of professional knowledge	PSS_Q14	0.909	0.014	66.994	0.909	*
	PSS_Q15	0.966	0.008	123.800	0.966	*
	PSS_Q16	0.931	0.008	115.710	0.931	*
Assignments and workload	PSS_Q9	0.721	0.029	24.533	0.721	*
	PSS_Q10	0.838	0.020	42.628	0.838	*
	PSS_Q11	0.806	0.022	36.677	0.806	*
	PSS_Q13	0.732	0.026	27.945	0.732	*
	PSS_Q12	0.793	0.023	33.808	0.793	*
Taking care of patients	PSS_Q1	0.684	0.025	27.072	0.684	*
	PSS_Q2	0.513	0.035	14.747	0.513	*
	PSS_Q3	0.662	0.029	22.920	0.662	*
	PSS_Q4	0.696	0.027	26.086	0.696	*
	PSS_Q5	0.693	0.029	23.704	0.693	*
	PSS_Q6	0.763	0.026	28.988	0.763	*
	PSS_Q7	0.547	0.034	16.004	0.547	*
	PSS_Q8	0.636	0.033	19.299	0.636	*
Clinical environment	PSS_Q17	0.784	0.028	27.838	0.784	*
	PSS_Q18	0.707	0.032	21.820	0.707	*
	PSS_Q19	0.572	0.036	15.670	0.572	*
Teachers and nursing staff	PSS_Q24	0.716	0.032	22.056	0.716	*
	PSS_Q25	0.662	0.034	19.305	0.662	*
	PSS_Q26	0.840	0.022	37.340	0.840	*
	PSS_Q27	0.580	0.035	16.699	0.580	*
	PSS_Q28	0.756	0.024	31.362	0.756	*
	PSS_Q29	0.704	0.028	25.438	0.704	*
Peers and daily life	PSS_Q20	0.600	0.032	19.007	0.600	*
	PSS_Q21	0.717	0.027	26.481	0.717	*
	PSS_Q22	0.763	0.027	28.409	0.763	*
	PSS_Q23	0.615	0.037	16.572	0.615	*

s.e., standard error; Sig., significance.

† , PSS, perceived stress scale.

* , *p* < 0.05.

There were also significant positive correlations amongst all six latent factors, indicating that participants who showed high perceived stress in one dimension were also likely to show high perceived stress in the other dimensions as well (see Table 6).

**TABLE 6:** Latent factor correlations.

Factor 1 (Stress from)†	Factor 2 (Stress from)†	Correlation	Sig.
Lack of professional knowledge	Assignments and workload	0.507	*
Lack of professional knowledge	Taking care of patients	0.738	*
Lack of professional knowledge	Clinical environment	0.625	*
Lack of professional knowledge	Teachers and nursing staff	0.575	*
Lack of professional knowledge	Peers and daily life	0.569	*
Assignments and workload	Taking care of patients	0.603	*
Assignments and workload	Clinical environment	0.635	*
Assignments and workload	Teachers and nursing staff	0.692	*
Assignments and workload	Peers and daily life	0.809	*
Taking care of patients	Clinical environment	0.662	*
Taking care of patients	Teachers and nursing staff	0.702	*
Taking care of patients	Peers and daily life	0.655	*
Clinical environment	Teachers and nursing staff	0.596	*
Clinical environment	Peers and daily life	0.680	*
Teachers and nursing staff	Peers and daily life	0.815	*

Sig., significance.

* , *p* < 0.05.

† , PSS, perceived stress scale.

Taken together, these results are consistent with previous studies showing a six-factor latent structure for the PSS (Sheu et al. 1997:341–351, 2002:165–175), consisting of stress from (1) taking care of patients, (2) teachers and nursing staff, (3) assignments and workload, (4) peers and daily life, (5) lack of professional knowledge and skills and (6) clinical environment, and the results confirm the construct validity of the PSS in this South African sample of 444 nursing students.

## Discussion

Whilst numerous international studies have focused on stress in nursing students, there are few validated instruments to measure stress (Watson et al. 2013:160), with the PSS by Sheu et al. (1997:341–351) being the most cited scale (Labrague et al. 2017:471–480; McCarthy et al. 2018:197–209). Despite the frequent use of the PSS, few studies have tested its validity, with most researchers referring to the original work done by the developers (Sheu et al. 1997:341–351, 2002:165–175) except for Algaralleh et al. (2019:777–787) who validated the Arabic version of the PSS amongst Jordanian nursing students. Based on a review of published literature, it appears as if the PSS has not been used in South Africa to determine perceived stress amongst nursing students. This is supported by the work of Langtree et al., who in 2018 noted the lack of an appropriate instrument to measure the causes of stress amongst first-year nursing students in South Africa and therefore developed their own scale.

Given the international popularity of the PSS and the dearth of research on the psychometric properties of the scale, this was the first study to measure the construct validity and internal consistency of the PSS for South African nursing students. The results confirmed the internal consistency and construct validity of the PSS for South African nursing students. More specifically, the six-factor model of the PSS identified in earlier research (Sheu et al. 1997:341–351, 2002:165–175) was confirmed. There were significant positive correlations amongst all six latent factors – (1) taking care of patients, (2) teachers and nursing staff, (3) assignments and workload, (4) peers and daily life, (5) lack of professional knowledge and skills and (6) clinical environment – indicating that participants who showed high perceived stress in one dimension were also likely to show high perceived stress in the other dimensions as well.

With regard to the internal consistency, alpha coefficients for the total scale and subscale scores were above the 0.70 cut-off, suggesting an acceptable degree of internal consistency (Taber 2018:1278). The alpha coefficient for the total scale was 0.93, which is higher than that found in other studies (Alsaqri 2017:3; Khater et al. 2014:196; Labrague 2013:426; Labrague et al. 2018:403; Sheu et al. 2002:168; Ugwoke et al. 2018:13214).

The overall mean score for the PSS was 1.48 (s.d. 0.61), which is indicative of moderate levels of stress. Similar findings were reported in studies conducted in Saudi Arabia (Ahmed & Mohammed 2019:120–121; Shdaifat, Jamama & Al Amer 2018:38) and Jordan (Khater et al. 2014:200–201). Higher scores, although still within the ‘moderate’ category, were reported in Saudia Arabia (Aedh et al. 2015:35; Alsaqri 2017:3), Turkey (Karaca et al. 2017:36), Philippines (Labrague 2013:427–428; Labrague et al. 2018:405), Greece (Labrague et al. 2018:405) and Nigeria (Labrague et al. 2018:405). Stress from assignments and workload was the main source of stress in the current study, whilst stress from lack of professional knowledge was the least reported. A possible explanation for this is that the survey was undertaken in the month prior to the commencement of examinations, when the students were finalising classes and assignments and not engaging in clinical work.

Increased levels of stress result in burnout which is correlated with poorer psychological and physical health and may influence the work of individuals in helping professions and eventually also the well-being of their patients (Enns et al. 2018:230). There is evidence that increased levels of stress and burnout in nursing students are negatively related to caring behaviours (Li et al. 2020:5–6); they also result in poorer job proficiency and nurses who are more inclined to leave the profession (Rudman & Gustavsson 2012:998; Rudman, Gustavsson & Hultell 2014:620). Given that nursing is already a highly stressful occupation (Dlamini & Visser 2017:1068; Khamisa et al. 2015:660; Van der Colff & Rothmann 2014:381) and the importance of retaining nurses in the profession (Van Rensburg 2014), stress should be addressed at the level of the nursing student. Therefore, the importance of valid and reliable scales to measure stress levels and sources of stress amongst nursing students should be acknowledged, so that meaningful and useful inferences can be made when recommending strategies to address stress in nursing students. The current study established that the PSS is a useful tool for nursing educators and researchers to help identify the main sources of stress amongst nursing students. As the nursing curriculum is both academically and clinically demanding, it is important for institutions providing nursing education to introduce preventative measures, such as teaching coping strategies and providing mentorship programmes, so that students are taught how to deal with stress at the outset of their education.

As with all research, this study has limitations. The study was undertaken at a single university amongst a convenience sample of nursing students, although slightly more than a third of the group was reached. As such, caution is required when generalising the findings to other university settings. A self-administered questionnaire was used, which implies that there may be some level of response bias. It is recommended that future research should replicate the present study and that further confirmatory factor analysis be undertaken to confirm the six-factor structure of the PSS for nursing students in South Africa.

## Conclusion

This study confirms the six-factor structure and internal consistency of Sheu et al.’s (1997:341–351) PSS, suggesting that it is a valid and reliable instrument to test the perceived stress from (1) taking care of patients, (2) teachers and nursing staff, (3) assignments and workload, (4) peers and daily life, (5) lack of professional knowledge and skills and (6) clinical environment for South African nursing students.
